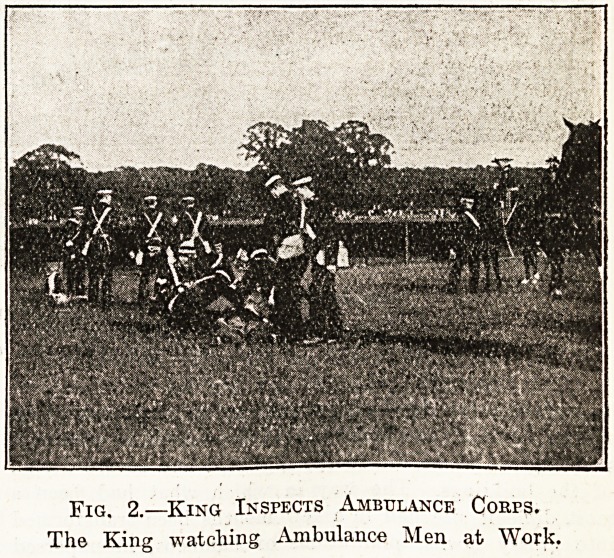# St. John's Brigade Reviewed in Windsor Great Park

**Published:** 1912-06-29

**Authors:** 


### THE KING AND AMBULANCE WORK.

### St. John's Brigade Reviewed in Windsor Great Park.

A review of the St. John's Ambulance Brigade was held in Windsor Great Park last Saturday, when his Majesty the King inspected more than 14,000 Brigade members and watched a life-saving display. The men were drawn up between the Long Walk and Queen Anne's Ride at the Windsor end, Major-General J. C. Dalton being in the post of honour.

In his inspection of the Brigade the King was accompanied by Colonel Sir James Clark, late

Fig. 1.—King Inspects Ambulance Corps.
Captain Aplin, R.N., Chief of Life-Saving Brigade. Captain Hudson on Right Front.

R.A.M.C., the Chief Commissioner, who was in command of the parade. A royal carriage followed, in which the Queen and the Prince of Wales were seated.

The first life-saving display showed saving from shipwreck by the Brigade members of Tynemouth and South Shields Volunteer Life Brigade, which was commanded by Captain Aplin, R.N., Assistant-Inspector, L.S.A., Board of Trade. The scene of the wreck was symbolised by a mast, and the rocket apparatus was soon in working order for the rescue of three men, who were visible in the crow's nest. A breeches-buoy was sent off, and the three men were promptly brought to the ground in safety, whereupon they were given over to the tender mercies of stretcher parties, who rendered first aid, as our illustration shows, and who finally bore them away to an improvised "hospital" in the form of a tent.

With distinctive appositeness, the second display exemplified ambulance and life-saving work on land and took the form of illustrating rescue work in mines. Mr. H. C. Chambers was in command of the Brigade members from various collieries, he himself being district secretary of District Number 5. A coal seam was reproduced on the other side of the ground, under the low roof of which a number of miners were discovered with their picks; and a flash of light simulated an explosion. The miners appeared to be overcome by the fumes, and the oxygen apparatus with which the rescuers were provided was one of the most interesting features of the display. Ever since last August mines, miners, and mining have occupied an increasing share of public attention, and the special form of first aid which this occupation requires is one which the general public may well appreciate.

This review is especially noteworthy from the fact that the King's interest in the work of the St. John's Ambulance Brigade is typical of his recent encouragement of the stricter forms of what is called preventive medicine. But that the ambulance side of this work is a strictly scientific and medically important one is readily illustrated by the striking article which we published last week on "Emergency Hospitals in Industrial Districts," which gave a good idea of this particular problem; and, further, can be proved by this review at Windsor, which should do much to focus public attention on the importance of an adequate provision of trained ambulance men and place the existing organisations on a still firmer and more openly dignified foundation.

Those who have had experience of practical first-aid teaching know how much energy, intelligence, and self-sacrifice are displayed by those who take up this work. They know how greatly keenness is sustained and augmented by two factors: one the appreciation and encouragement of those in high places, and of the general public; and the other,

Fig. 2.—King Inspects Ambulance Corps.
The King watching Ambulance Men at Work.

an instructor who takes his class in a thoroughly serious and earnest vein, and provides them, as far as it may be within his power, with opportunities of seeing in practice the application of the principles which he has inculcated into them theoretically. In this respect displays such as that of last Saturday have an immense value.

## Figures and Tables

**Fig. 1. f1:**
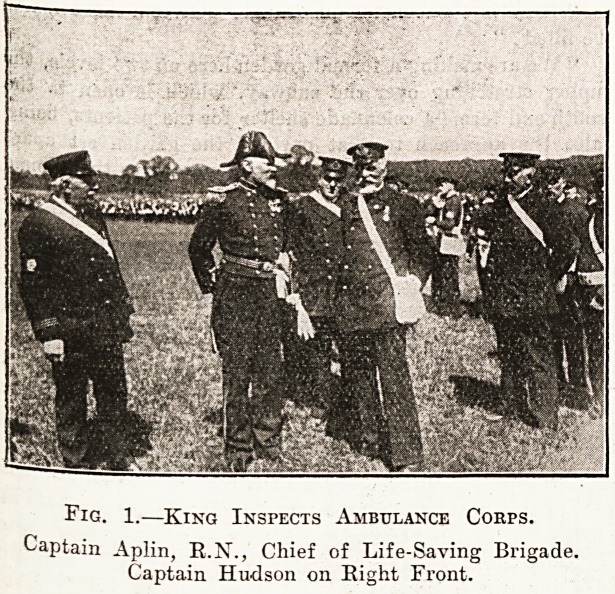


**Fig. 2. f2:**